# Supplementary material for: A comparison of two remotely operated vehicle (ROV) survey methods used to estimate fish assemblages and densities around a California oil platform
Source: PLoS One. 2020 Nov 10;15(11):e0242017. doi: 10.1371/journal.pone.0242017 (PMC7654814; doi:10.1371/journal.pone.0242017)
Supplement: S1 File — (DOCX) [file pone.0242017.s001.docx]

Love, M., M. Nishimoto, and L. Kui. 2020. Data to support manuscript: A Comparison of Two ROV Survey Methods Used to Estimate Fish Assemblages and Densities Around a California Oil Platform ver 1. Environmental Data Initiative. <https://doi.org/10.6073/pasta/5f2d77235388717672ff612cc7fa7d7c>.
