# Supplementary material for: A comparison of two remotely operated vehicle (ROV) survey methods used to estimate fish assemblages and densities around a California oil platform
Source: PLoS One. 2020 Nov 10;15(11):e0242017. doi: 10.1371/journal.pone.0242017 (PMC7654814; doi:10.1371/journal.pone.0242017)
Supplement: Attachment — Submitted filename: Response to Reviewers.22 September 2020.docx [file pone.0242017.s003.docx]

22 September 2020

Editor

PLOSONE

This is the final version of:

A Comparison of Two Remotely Operated Vehicle (ROV) Survey Methods Used to Estimate Fish Assemblages and Densities Around a California Oil Platform

Love, M. S., M. M. Nishimoto, S. Clark, L. Kui, A. Aziz, D. Palandro

In response to the comments of reviewer #2:

1) I have changed the abstract, so that it is only a single paragraph.
2) I have removed the comma after “particularly” in line 177.

Milton Love

Research Biologist

Marine Science Institute

University of California

Santa Barbara, California
